# The effects of common structural variants on 3D chromatin structure

**DOI:** 10.1186/s12864-020-6516-1

**Published:** 2020-01-30

**Authors:** Omar Shanta, Amina Noor, Human Genome Structural Variation Consortium (HGSVC), Mark J. P. Chaisson, Ashley D. Sanders, Xuefang Zhao, Ankit Malhotra, David Porubsky, Tobias Rausch, Eugene J. Gardner, Oscar L. Rodriguez, Li Guo, Ryan L. Collins, Xian Fan, Jia Wen, Robert E. Handsaker, Susan Fairley, Zev N. Kronenberg, Xiangmeng Kong, Fereydoun Hormozdiari, Dillon Lee, Aaron M. Wenger, Alex R. Hastie, Danny Antaki, Thomas Anantharaman, Peter A. Audano, Harrison Brand, Stuart Cantsilieris, Han Cao, Eliza Cerveira, Chong Chen, Xintong Chen, Chen-Shan Chin, Zechen Chong, Nelson T. Chuang, Christine C. Lambert, Deanna M. Church, Laura Clarke, Andrew Farrell, Joey Flores, Timur Galeey, Madhusudan Gujral, Victor Guryev, William Haynes Heaton, Jonas Korlach, Sushant Kumar, Jee Young Kwon, Ernest T. Lam, Jong Eun Lee, Joyce Lee, Wan-Ping Lee, Sau Peng Lee, Shantao Li, Patrick Marks, Karine Viaud-Martinez, Sascha Meiers, Katherine M. Munson, Fabio C. P. Navarro, Bradley J. Nelson, Conor Nodzak, Amina Noor, Sofia Kyriazopoulou-Panagiotopoulou, Andy W. C. Pang, Gabriel Rosanio, Mallory Ryan, Adrian Stütz, Diana C. J. Spierings, Alistair Ward, Anne Marie E. Welch, Ming Xiao, Wei Xu, Chengsheng Zhang, Qihui Zhu, Xiangqun Zheng-Bradley, Ernesto Lowy, Sergei Yakneen, Steven McCarroll, Goo Jun, Li Ding, Chong Lek Koh, Paul Flicek, Ken Chen, Mark B. Gerstein, Pui-Yan Kwok, Peter M. Lansdorp, Gabor T. Marth, Jonathan Sebat, Xinghua Shi, Ali Bashir, Kai Ye, Scott E. Devine, Michael E. Talkowski, Ryan E. Mills, Tobias Marschall, Jan O. Korbel, Evan E. Eichler, Charles Lee, Jonathan Sebat

**Affiliations:** 1 Department of Electrical and Computer Engineering, UCSD, San Diego, CA USA; 2 Beyster Center for Genomics of Psychiatric Diseases, Department of Psychiatry, UCSD, San Diego, CA USA; 3 Department of Cellular and Molecular Medicine, UCSD, San Diego, CA USA; 4 Department of Pediatrics, UCSD, San Diego, CA USA

**Keywords:** Hi-C, Structural variation, Deletion, Inversion, TAD, TAD fusion, Chromatin

## Abstract

**Background:**

Three-dimensional spatial organization of chromosomes is defined by highly self-interacting regions 0.1–1 Mb in size termed Topological Associating Domains (TADs). Genetic factors that explain dynamic variation in TAD structure are not understood. We hypothesize that common structural variation (SV) in the human population can disrupt regulatory sequences and thereby influence TAD formation. To determine the effects of SVs on 3D chromatin organization, we performed chromosome conformation capture sequencing (Hi-C) of lymphoblastoid cell lines from 19 subjects for which SVs had been previously characterized in the 1000 genomes project. We tested the effects of common deletion polymorphisms on TAD structure by linear regression analysis of nearby quantitative chromatin interactions (contacts) within 240 kb of the deletion, and we specifically tested the hypothesis that deletions at TAD boundaries (TBs) could result in large-scale alterations in chromatin conformation.

**Results:**

Large (> 10 kb) deletions had significant effects on long-range chromatin interactions. Deletions were associated with increased contacts that span the deleted region and this effect was driven by large deletions that were not located within a TAD boundary (nonTB). Some deletions at TBs, including a 80 kb deletion of the genes CFHR1 and CFHR3, had detectable effects on chromatin contacts. However for TB deletions overall, we did not detect a pattern of effects that was consistent in magnitude or direction. Large inversions in the population had a distinguishable signature characterized by a rearrangement of contacts that span its breakpoints.

**Conclusions:**

Our study demonstrates that common SVs in the population impact long-range chromatin structure, and deletions and inversions have distinct signatures. However, the effects that we observe are subtle and variable between loci. Genome-wide analysis of chromatin conformation in large cohorts will be needed to quantify the influence of common SVs on chromatin structure.

## Background

3D chromatin structure is characterized by Topologically Associated Domains (TADs) and chromatin loops, which create physical interactions between genes and distant regulatory sequences [1]. CTCF and the protein complex cohesin are localized to the boundaries of TADs [2–4], where they serve as barriers to the spread of chromatin. Genetic variation in these sequences has the potential to influence the binding of these factors and contribute to variability in chromatin structure in humans. However, little is known about patterns of topological variation in the population and the underlying genetic mechanisms.

Structural Variants (SVs) are a major source of genetic variability, and SVs have significant functional impact on the genome through the deletion or rearrangement of coding and regulatory sequences. Notably, large SVs that disrupt or re-establish chromatin contacts are associated with two rare monogenic disorders including human limb malformations [5–7] and female-to-male sex reversal [5]. Multiple recent studies have begun to examine the potential of SVs to influence chromatin conformation by theoretical modeling of ChIA-PET [8] or Hi-C [9] data from a single cell line (GM12878). However, these studies have not directly investigated how genetic variation between individuals contributes to variation in large-scale chromatin structure.

In this study, we investigated the effect of common SV polymorphism on 3D chromatin structure in a sample of individuals from the 1000 genomes project [10]. Specifically we sought to test the hypothesis that deletions of the boundary regions between adjacent TADs could result in large scale alterations in chromatin conformation. We performed Chromatin Conformation Capture (Hi-C) sequencing of lymphoblastoid cell lines (LCLs) of 19 individuals from the 1000 genomes project, and we tested the effects of common SVs on the numbers of nearby chromatin contacts.

## Results

**Fig. 1:**
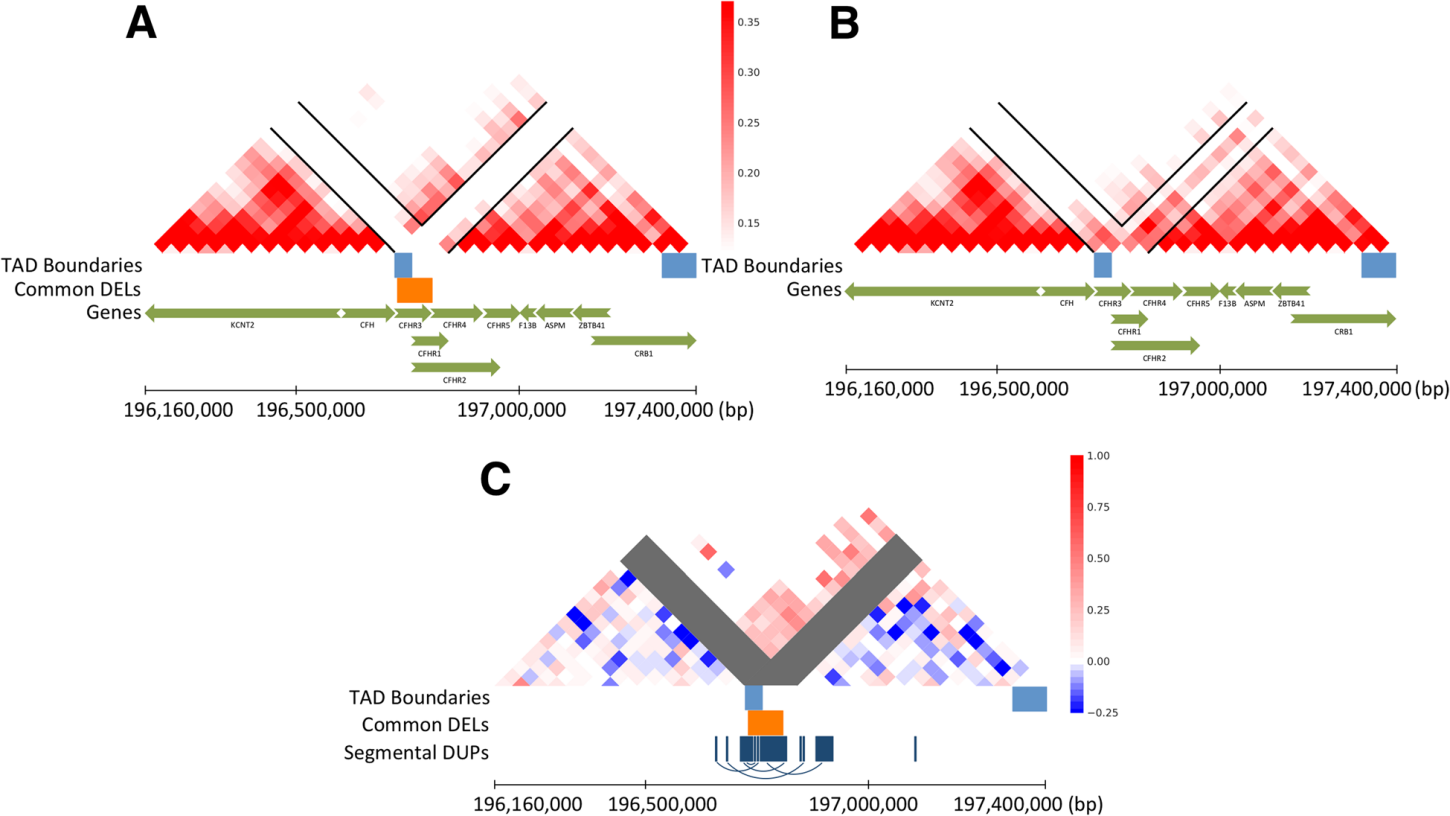
Deletion of CFHR3 and CFHR1 is associated with variation in chromatin conformation. Maps of chromatin interaction surrounding an 80 kb deletion of the CFHR3 and CFHR1 genes (hg19 position chr1:196,728,877–196,808,865) are depicted by averaging the counts within the contact matrices of subjects homozygous for the deletion haplotype (*N* = 3, Panel **a**) and subjects homozygous for the reference haplotype (*N* = 12, Panel **b**). Normalized counts were plotted as a heatmap with red tone representing the number of chromatin interactions in 40 kb bins. To better visualize the effects for this example, the correlation of counts with the deletion haplotype was tested for all bins across a 1.24 Mb region by linear regression, and regression coefficients were displayed as a blue-red heatmap (Panel **c**)

We hypothesize that SVs could influence TAD structure *indirectly* by disrupting regulatory sequences that control formation of TADs in adjacent genomic regions. In addition, we anticipate that SVs will have *direct* effects on the coverage and spacing of paired-end reads similar to the effects that are ordinarily observed for SVs in whole genome sequence data [11]. We sought to distinguish these two types of effects by separately quantifying the direct effects on chromatin interactions that span a deletion breakpoint and indirect effects on chromatin interactions adjacent to a deletion. We illustrate this with an example in Fig. 1; a large deletion of ~ 80 kb that disrupts the complement factor H-related genes CFHR3 and CFHR1. This deletion has been associated with decreased risk of age-related macular degeneration (AMD), an increased risk of atypical hemolytic uremic syndrome (aHUS), and systemic lupus erythematosus (SLE) [12–15]. A map of chromatin contacts for the deleted region and two adjacent TADs (spanning 1.24 Mb) is illustrated in Fig. 1 at a 40 kb resolution. The average number of contacts is shown for subjects who were homozygous for the deletion (Fig. 1 a) and for subjects who were homozygous for the reference allele (Fig. 1 b). As expected, the deletion results in loss of contacts in bins that overlap with the deleted region, and as adjacent regions are brought closer together, we observe an increase in contacts that span the deletion.

The regional effects of the CFHR3/1 deletion on TAD structure was examined in more detail by correlating counts with genotype for all elements of the contact matrix using linear regression controlling for ancestry and sex. The resulting correlation matrix is visualized as a heatmap of the regression coefficients (Fig. 1 c, see methods). The correlation matrix reveals a pattern consistent with an increase in interactions between the proximal TAD (involving the CFH gene) and the distal TAD (involving a broad region between the genes CFHR2 and CRB1). A portion of the CFHR3/1 deletion overlaps with multiple annotated segmental duplications (SDs) which could potentially confound the mapping of Hi-C read pairs. A similar analysis was conducted after masking segmental duplications and the observed effects were unchanged. Therefore, the effects we observe are not explained by the segmental duplications or by contacts between paralogous sequences. Furthermore, a map of SDs across the region (Fig. 1 c) shows that the positive effects that span the deletion primarily involve contacts between heterologous sequences.

**Fig. 2:**
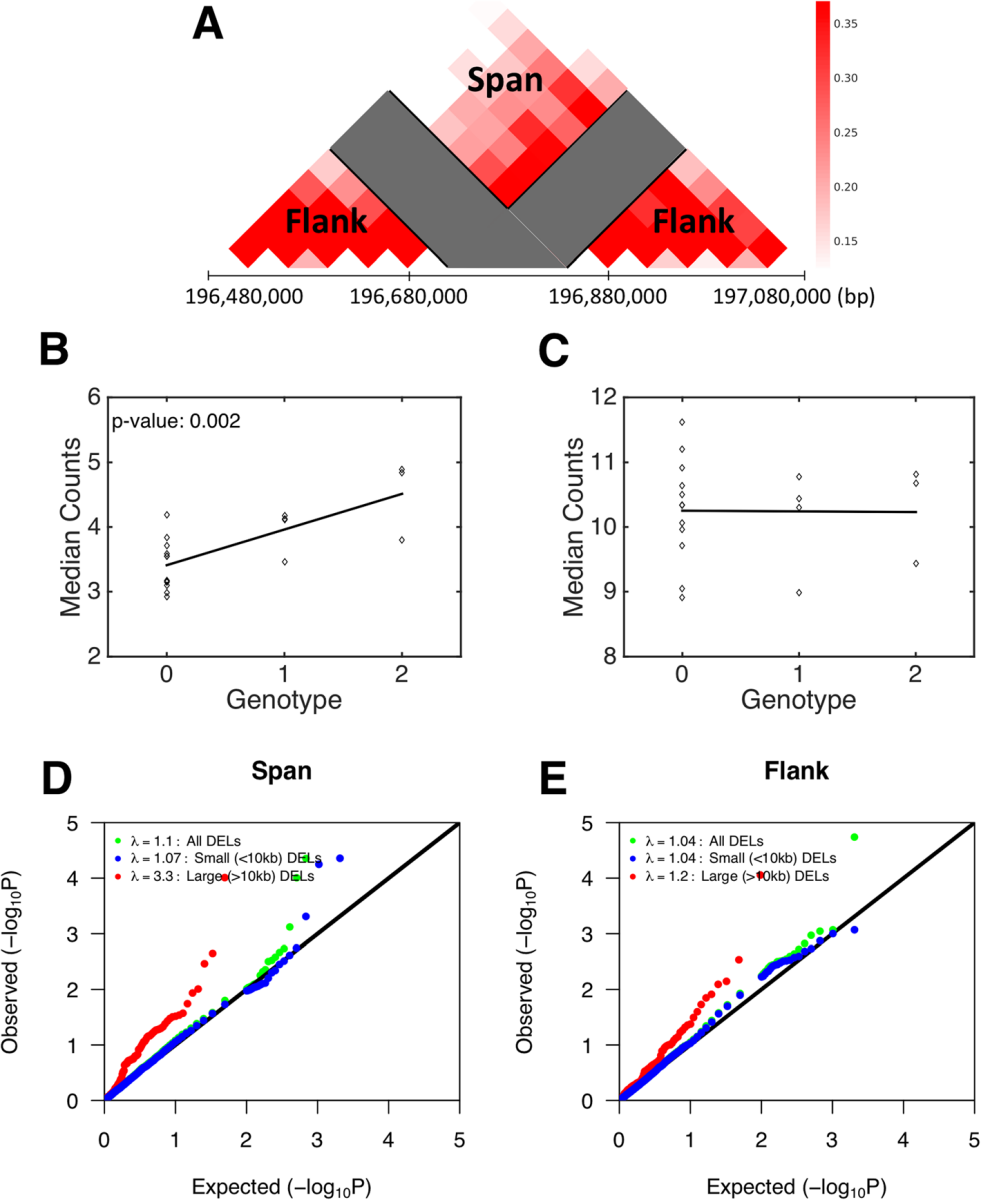
Testing the effect of common deletions on chromatin conformation. The Hi-C map of chromatin interactions for the 80 kb CFHR3/1 deletion was separated into regions that interact across the deletion (span) and regions that do not cross the deletion (flank) as they can exhibit different behavior with the removal of the deletion bins (Panel **a**). The effect of the deletion on chromatin conformation was investigated by linear regression, showing a significant effect in the span region (*p*-value: 0.002, Panel **b**) and no effect in the flank region (Panel **c**). The same analysis was run for all common deletions and *p*-values stratified by size at a 10 kb threshold were displayed in a QQ plot. Large deletions have the strongest effect in the span region while the contribution from small deletions is non-existent (Panel **d**). Large deletions show a smaller effect in the flank region (Panel **e**)

To more rigorously determine the association of deletions with chromatin conformation, we used a linear regression model to test for the effects of deletions on chromatin contacts. We again use the CFHR3/1 example to illustrate (Fig. 2). Counts were averaged for elements that span the deletion and for flanking regions within 240 kb (Fig. 2 a), a region chosen as the optimal distance by a parameter sweep (see methods). The effects of deletions on chromatin conformation were then tested for “span” and “flank” separately by linear regression controlling for ancestry principal components (PCs) and sex. Other potential confounders were evaluated, including surrogate variables, to account for unknown sources of noise (see methods), however including these additional covariates did not reduce the overall inflation of the test statistic (Additional file 1: Fig. S1). The effect of the CFHR3/1 deletion on spanning contacts was statistically significant (Fig. 2 b, p-value: 0.002), but the deletion did not have a significant effect on the number of contacts in the flanking regions that overlap with the adjacent TADs (Fig. 2 c).

We next sought to extend the analysis of Hi-C data to all common deletions in the phase 3 release of the 1000 genomes project [10]. Analysis was restricted to all deletions that were present in ≥ 3/19 samples (*N* = 2180 deletions). The deletions ranged in size from 51 bp to 125 kb, with an average size of 2622 bp. The magnitude of the genetic effects was assessed based on genomic inflation of the test statistic (λ). A Quantile-Quantile (QQ) plot of observed regression *p*-values relative to an empirical null distribution based on permutation of genotypes shows very modest effects for deletions overall, λ = 1.10 and 1.04 for span (Fig. 2 d) and flank (Fig. 2 e) respectively, but the effects were stronger for large (> 10 kb) deletions (λ = 3.30 and 1.20 for span and flank respectively). The magnitude of the effect of large deletions on the spanning contacts was greater than for small deletions (Kolmogorov-Smirnov test, p-value: 7.63 × 10^− 6^), but was not significantly different for the flank region (p-value: 0.132). Summary statistics for all deletions that were tested are included in Additional file 2: Table S1. Given that the effects of common deletions on chromatin conformation are driven by large deletions, our subsequent analyses focused on this subset of SVs.

**Fig. 3:**
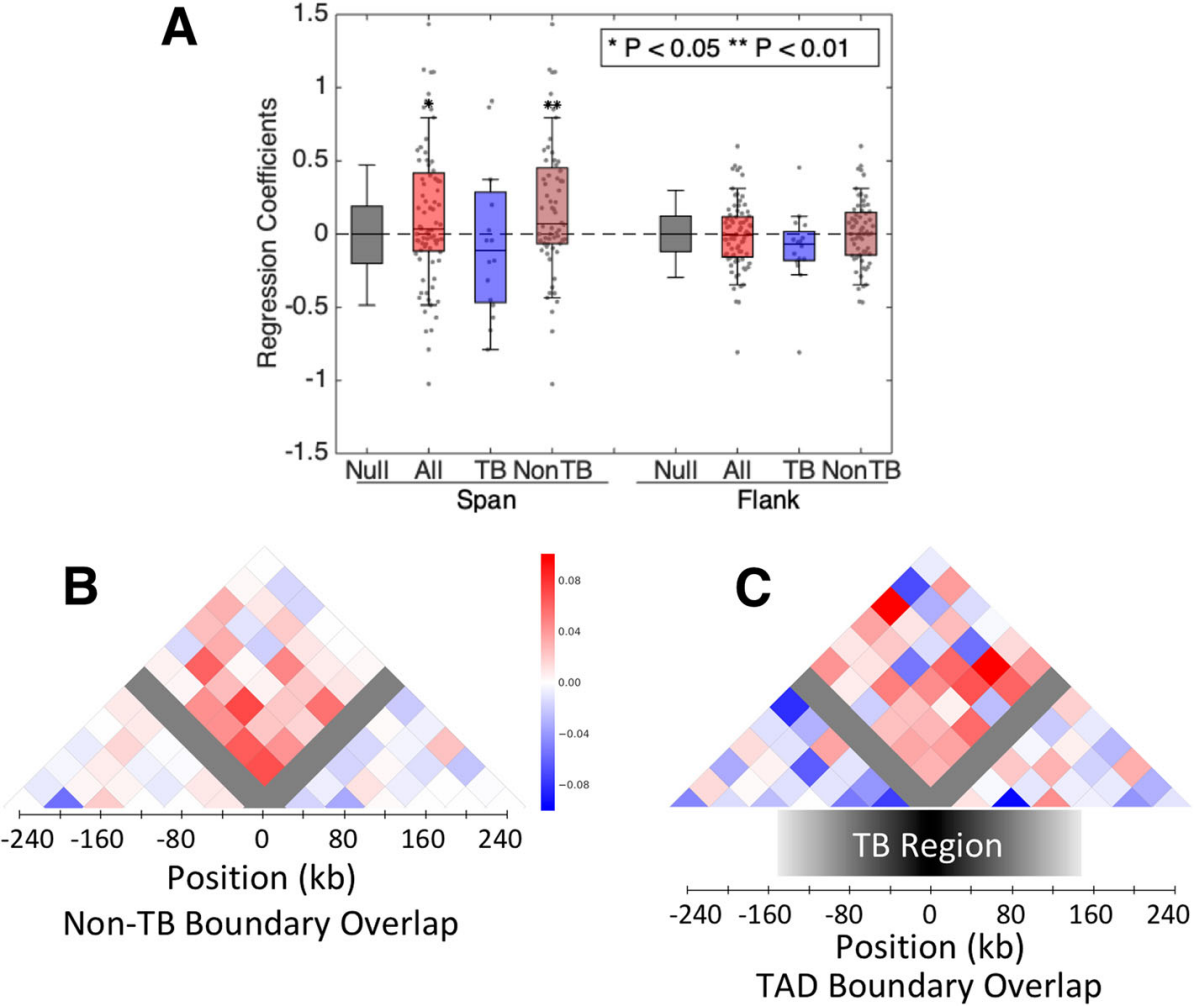
Large deletions that do not intersect a TAD boundary have a significant positive effect on the number of contacts that span the deletion region. To determine if the strength or direction of effects differed for deletions located at the boundaries of TADs, regression coefficients from our genome wide analysis were compared between groups of deletions located at TAD boundaries (TB) and those not at TAD boundaries (NonTB) (Panel **a**). A Wilcoxon rank-sum test was performed for each group against a null distribution, resulting in a significant positive effect for the span region of NonTB deletions (p-value: 0.002). To visualize the topological changes of these effects, a blue-red heatmap of regression coefficients was constructed for NonTB and TB deletions separately. A linear regression was performed for each pairwise bin interaction and coefficients were averaged across deletions. Deletions not present at TAD boundaries have positive values in the span region (Panel **b**). Deletions that intersect TAD boundaries do not have a unique trend in the span or flank region (Panel **c**)

TAD boundaries correlate with insulator and barrier elements that control chromatin conformation and gene regulation [2]. We therefore hypothesized that deletions could have more dramatic effects on chromatin conformation when they occur in TAD boundaries. Common large deletions (*N* = 80 deletions) were separated into deletions at TAD boundaries (TB, *N* = 16 deletions) and those not at a TAD boundary (NonTB, *N* = 64 deletions). The distribution of regression coefficients for common large deletions in TB/NonTB categories was compared against an empirical null distribution based on permutation of genotypes. These results show a statistically significant positive effect for the span region of NonTB deletions (Wilcoxon rank-sum test, *p*-value: 0.002) (Fig. 3 a). A visualization of the change in chromatin structure is illustrated by averaging each element of the contact matrix within 240 kb of a deletion across loci in TB/NonTB categories separately (Fig. 3b, c). For NonTB deletions we observe an increase in the number of deletion spanning contacts (Fig. 3a) that is concentrated within a narrow region around the deletion (Fig. 3b). This pattern is consistent with the “direct” effects of deletion on the number of breakpoint-spanning read pairs. We do not see a significant effect of NonTB deletions on the number of contacts within the adjacent flanking regions. For TB deletions, we did not detect significant effects on the number of spanning or flanking contacts (Fig. 3a). These results suggest that TB deletions have effects that are relatively subtle or that are quite variable between loci, but studies of larger samples would be needed to determine if effects differ consistently between TB and nonTB deletions. Analysis was repeated after masking segmental duplications and results were unchanged (Additional file 3: Fig. S2).

A recent paper has described a method to predict the potential of deletions to cause the fusion of two adjacent TADs [9], a potential mechanism described in [16]. This study reported that deletions at TAD boundaries are under negative selection and deletions with a high “fusion score” were skewed toward a low frequency. Using the deletion-spanning contacts for 80 large common deletions as a measure of TAD fusion, we examined whether there was a correlation between the fusion score of the deletion and the coefficient from the regression. We found no correlation of the predicted fusion scores with the observed effects of these deletions on spanning contacts (Additional file 4: Fig. S3).

**Fig. 4:**
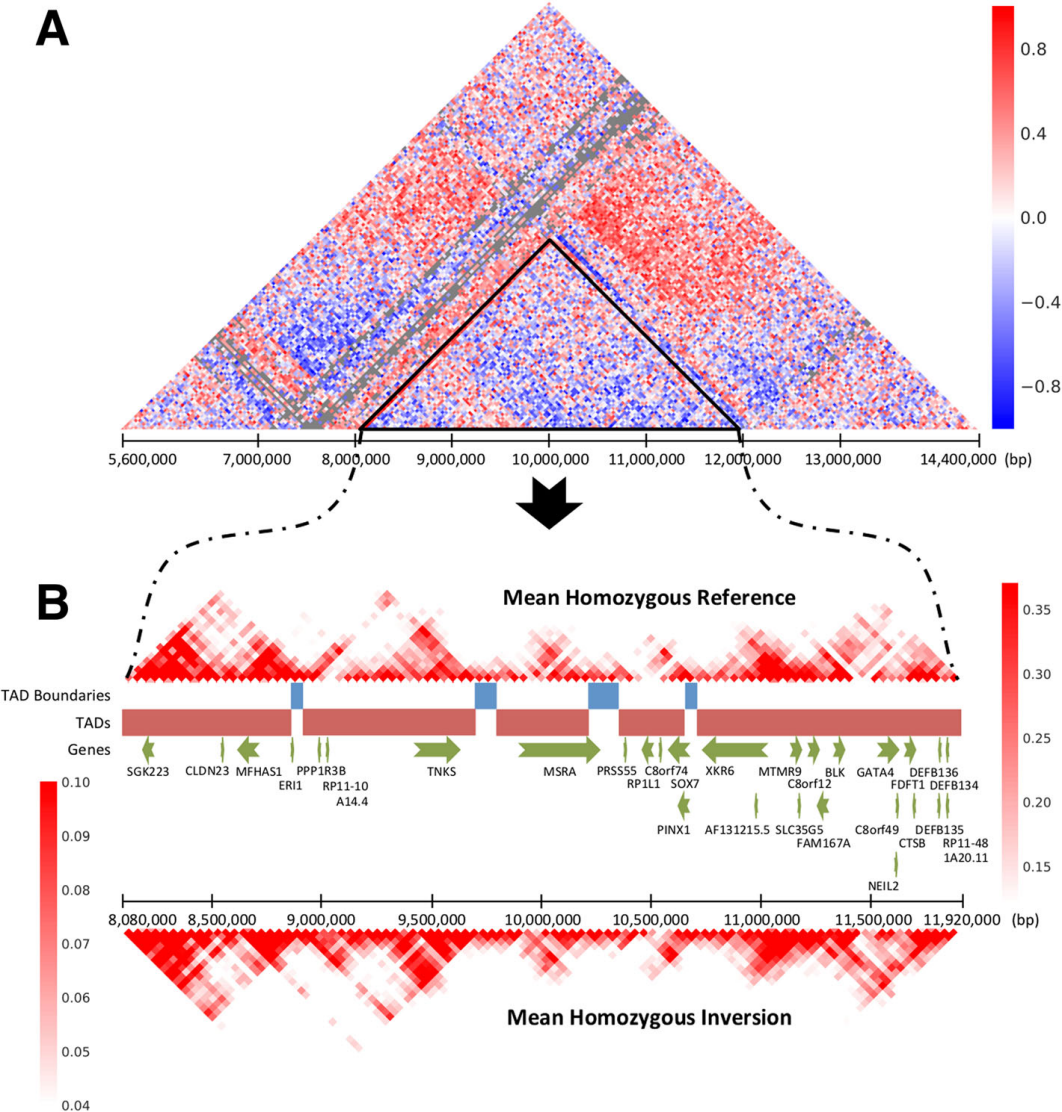
Long range effects of a large 8p23 inversion on chromatin conformation. A correlation heatmap shows chromatin interactions that are gained (red) and lost (blue) on the inversion haplotype relative to the reference (Panel **a**). The gray region corresponds to missing values that could not be normalized. The inversion region is depicted by the black triangle. Hi-C matrices for samples that were homozygous for the absence of an inversion and homozygous for the inversion at 8p23.1 were averaged separately and annotated (Panel **b**). The TAD structure is preserved in a mirrored fashion along with their associated genes. Chromatin interactions for the inversion were mirrored to aid visual comparison with the reference

Our results suggest that large SVs have detectable effects on chromatin conformation. Since the above analysis focused on deletions, it did not assess the largest common SVs known to exist in the population, which include large inversions of 8p23.1 (3.87 Mb) and 7q11.1 (2.45 Mb). To characterize the effects of large inversions on chromatin conformation, inversion genotypes were obtained from single-cell strand sequencing (Strand-seq) of a subset of 9 subjects in the 1000 genomes project [17], and the correlation of chromatin contacts across the region was visualized (Fig. 4 a). The most dramatic effects of the inversion involve contacts that span the inversion breakpoints, denoted by the black triangle, and these effects span distances > 2 Mb from the breakpoint.

The availability of a full assembly of the 8p23.1 inversion haplotype [18] enabled us to map TAD structure of the inversion haplotype by directly mapping Hi-C data of subjects that were homozygous for the 8p23.1 inversion to the inversion haplotype. The average number of contacts is shown for subjects with homozygous genotypes for the inversion (Fig. 4 b, bottom) and the reference haplotype (Fig. 4 b, top). TAD structures of the reference and inversion haplotypes were similar, and the same 5 TADs were defined. Patterns of long-range contacts for the inversion of 7q11.1 were similar (Additional file 5: Fig. S4).

We hypothesize that the genetic variants that influence chromatin conformation could thereby influence gene regulation [19]. However, the effects detectable in our current dataset are restricted to large SVs, relatively few of which represent lead variants for expression quantitative trait loci (eQTLs). Of the 2180 common deletions from our analysis and 5128 SV-eQTLs that were previously identified in another study [20], 75 common deletions tested in this study correspond to SV-eQTLs, and these were larger on average with an average length of 5.98 kb compared to the rest of the 2105 deletions which had an average length of 2.5 kb. A Wilcoxon rank sum test was performed between these two groups to determine if there was a significant difference between the regression *p*-value distribution of the deletions with SV-eQTLs and the regression p-value distribution of deletions without SV-eQTLs in the span region. However, SVs that were driving eQTLs did not have stronger effects on chromatin contacts (p-value: 0.45). Summary statistics for all deletions are annotated with SV-eQTLs in Additional file 2: Table S1.

## Discussion

Hi-C has enabled discoveries related to understanding the structural and functional basis of the genome. We show that large common deletions have significant effects on patterns of chromatin conformation with effects that are sufficiently large to be detectable in our small sample of 19 subjects.

Large common deletions have a distinctive signature characterized by positive effects on contacts that span the deletion. The most dramatic example was a common deletion polymorphism at CFHR3/1, which results in the gain of contacts that span a broad region betweem two adjacent TADs. An increase in the number of contacts between two distinct TADs is an effect reminiscent of “TAD fusion” [21] (Fig. 1). However, for most large common deletions, their effects on the number of deletion-spanning contacts were more subtle and were concentrated within a narrow region around the deletion (Fig. 3 b).

The effect of common SVs on 3D chromatin conformation has potential significance for gene regulation. However, in our current sample size, we are only able to capture effects from the largest and most common SVs, few of which are associated with expression QTLs.

Our results are consistent with common SVs having signatures in Hi-C data that are distinguishable but subtle. We reason that common SVs might tend to have relatively small effects on TAD structure as compared to rare pathogenic variants that have been described previously [5–7]. Deletions that remove TAD boundaries and cause TAD fusion may be under negative selection in the population and would therefore tend to be rare. Well-powered characterization of the effects of SVs on chromatin structure and gene regulation would therefore require Hi-C characterization of common variants in larger samples combined with targeted Hi-C and RNA sequencing of patient samples with specific rare disease associated variants.

Large common inversions have distinct effects on chromatin interactions that span the inversion breakpoints, and these effects can extend for distances > 2 Mb. TAD structures within the large inverted segments of two common inversions appear to be well preserved, suggesting that the sequences within the inverted regions are sufficient to determine their 3D structures.

## Conclusions

Our analysis has shown that large common SVs can influence local 3D chromatin structure, and the strength and direction of the observed effect varies by locus. Deletions and inversions have distinct signatures. Deletions increase the amount of chromatin interaction between adjacent regions while inversions rearrange the contacts that span its breakpoints.

## Methods

### Generation of hi-C data for 19 subjects

Hi-C data was generated for 19 subjects from the 1000 Genomes Project (Additional file 2: Table S1) using a “dilution” HindIII protocol as previously described [1]. Data collection is described in detail within a companion manuscript [22]. Hi-C allows for unbiased identification of chromatin interactions by using the following process: cells are cross-linked with formaldehyde, DNA is digested using the HindIII restriction enzyme that leaves a five-prime overhang, the five-prime overhang is filled with nucleotides, the resulting fragments are ligated under dilute conditions, DNA is sheared and fragments containing biotin are identified by paired-end sequencing [1]. Read ends were aligned to hg19 with BWA-MEM v0.7.8 [23] and in the case of split alignments, the five-prime-most alignment was used as the primary alignment. Reads without a five-prime end alignment and alignments with low mapping quality were filtered out. WASP was used to generate alternative reads and realigned using the BWA-MEM [24, 25]. Reads that did not have all alternative reads aligned to the same location were removed. Reads were re-paired and valid read pairs were pairs in which both reads passed this filtering.

Contact matrices were generated and normalized by dividing read pairs into 40 kb bin pairs and normalizing raw counts using HiCNorm [26, 27]. To compare matrices across samples, we needed to remove unwanted variation between matrix elements due to date of processing as well as remove any other batch effects. This was corrected for by using Bandwise Normalization and Batch effect Correction (BNBC, preprint on bioRxiv https://www.biorxiv.org/content/10.1101/214361v1). This method involves performing quantile normalization on a matrix that contains all contacts between loci at a fixed genomic distance.

### Defining TAD boundaries

TADs were defined as follows. Directionality Index (DI) was computed for each 40 kb bin and used in a Hidden Markov Model to predict the probability of a bin being upstream bias, no bias, or downstream bias [2]. TAD boundaries were called as regions switching from upstream bias to downstream bias.

### Extracting structural variant regions from the hi-C contact matrix

Genotypes for 68,818 SVs were obtained on the same subjects from the phase 3 SV calls from the 1000 genomes project [10]. The phase 3 SV call set includes 42,279 deletions, 6,025 duplications and 20,514 inversion/insertion/complex SVs, of which 5,517 deletions, 101 duplications, and 227 inversion/insertion/complex SVs were present at least once in our sample of 19 subjects. Given that deletions vastly outnumber all other classes of variants, we focused our primary analysis on these. Only deletion alleles that were present in ≥3/19 subjects (*N* = 2180 deletions, Additional file 2: Table S1) were included in our analysis. Deletions were then mapped to 40 kb bins within the chromosome Hi-C contact matrices. The bins of the contact matrix that “span” or “flank” each deletion were then defined as illustrated in Fig. 2. To determine the flanking distance that optimally captures the effect of deletions on flanking regions, multiple bin sizes were tested by a parameter sweep. Effects weakened as the distance increased from the deletion and 6 flank bins displayed the largest effect.

### Quantifying effects of common deletions on TAD structure

Quantitative effects of deletions on chromatin conformation were tested by Ordinary Least Squares Regression (OLSR) using Python. First, bins that overlapped with SVs were masked and specific deletion-flanking and deletion-spanning target regions were defined within 240 kb (six 40 kb bins) on either side of the deletion (Fig. 2 a). For each sample, contacts were averaged across the flanking and spanning target regions respectively. Regression was performed for each deletion on the span and flank regions separately, controlling for ancestry PCs obtained from SNP genotypes using PLINK1.9 software [28] and sex. The regression was constructed with normalized chromatin interaction counts between regions near the deletion as the independent variable and copy number as the dependent variable (0: Homozygous reference, 1: Heterozygous deletion, 2: Homozygous deletion).

### Selection of covariates used in regression model

The genomic inflation factor (λ) was used to determine how much of the effect could be attributable to confounding variables such as ethnicity or other unobserved noise in the data that could be captured with surrogate variables. Covariate terms were added one at a time and λ was calculated for the span and flank regions after each addition (Additional file 1: Fig. S1A). The possible confounding variables tested include ancestry PCs to control for population stratification, sex, and surrogate variable PCs to control for variation within each chromosome. Given the sample size of 19, the model becomes saturated with more than two variables [29]. Covariates were chosen, according to the combination that minimized λ. The lowest inflation included two ancestry PCs and sex as covariates. The proportion of variance explained by the first two ancestry PCs was calculated to be 47%. The ancestry PC and sex model was used for the rest of the study and regression coefficients for all loci were displayed in a boxplot (Fig. 3 a).

### Visualization of topological effects for CFHR3/1 and across multiple loci

Effects were visualized for select loci as heatmaps of regression coefficients. Each heatmap is constructed by applying the regression model for all bins separately across a target genomic region. To visualize the topological effect for CFHR3/1, the regression coefficients for each bin were then plotted as a heatmap with red indicating positive correlation, blue indicating negative correlation, and bins that overlapped the deletion were indicated in gray (Fig. 1 c).

In addition, to visualize “average” effects across multiple loci, matrices were centered on the left and right deletion boundaries, and the median regression coefficient for each bin across multiple loci was displayed as a heatmap (Fig. 3 b and c).

### Analysis of large inversions

Hi-C chromatin interactions for the bins that overlap the inversion and 62 bins on each side of the inversion were extracted. A Pearson correlation between number of chromatin interactions and genotype was applied for each bin across the 9 samples that had both Hi-C data and inversion calls available. The Pearson correlation for each bin was displayed as a heatmap (Fig. 4 a).

### Annotation of structural variants with summary statistics and eQTLs

All 2180 common deletions were first annotated with summary statistics from the regression analysis by reporting a *p*-value and regression coefficient describing the effect of the variant on both the flank region and span region. The SVs were then intersected with the TAD boundaries previously defined in the methods and defined as overlapping that TAD boundary if the intersection was at least 1 bp. An empty element in the table represents no overlap with a TAD boundary. All deletions were intersected with SV-eQTLs previously identified in another study [20]. If these SV-eQTLs were also present within the GWAS Catalog [19], then the table was further annotated with gene information like gene name, gene ID, etc.

## Supplementary information

**Additional file 1: Figure S1.** Ancestry principal components and sex need to be used as covariates in linear regression. To determine which covariates reduce the bias in the linear regression model, the effect of common deletions on chromatin conformation was tested for 6 different models, with each model adding an extra covariate term (Panel A). The genomic inflation factor was the metric used to determine the model with the least bias for possible confounding variables: ancestry principal components (PCs), sex, and surrogate variable PCs. The model that used ancestry PCs and sex as covariates had the least bias (λ = 1.10,1.04) and was chosen as the optimal model. *P*-values of the regression for each deletion in the span (Panel B) and flank (Panel C) region display how the chosen model still has inflation despite the low genomic inflation factor that can be attributed to real effects

**Additional file 2: Table S1.** Summary statistics for all common deletions. 2180 common deletions from 19 individuals in the 1000 Genomes Project were annotated with TAD boundaries, eQTLs, and GWAS hits. To investigate the effect of these deletions on chromatin conformation, a linear regression was performed between genotype and the median number of chromatin interactions within the flank and span region of each deletion. Ancestry principal components and sex were used as covariates in the regression model

**Additional file 3: Figure S2.** Masking segmental duplications does not change the effects of deletions on chromatin conformation. To determine if the effects on chromatin conformation are driven by segmental duplications (SD), a separate analysis was conducted for all large common deletions after masking every SD found within the deletion or in the flank regions. Deletions were stratified into groups of those that overlap with TAD boundaries (TB) and those that do not overlap with TAD boundaries (NonTB). A Wilcoxon rank-sum test was performed for each group against a null distribution and the results are consistent with the analysis that did not involve SD masking, showing that the effects of deletions on chromatin contacts are not driven by segmental duplications

**Additional file 4: Figure S3.** Linear regression coefficients in the span region do not correlate with TAD fusion score. We generated the TAD fusion score for our 80 large common deletions and compared the result with the linear regression coefficients in the span region. There was no significant correlation between the two different methods; suggesting that the fusion score is not predictive of patterns of chromatin conformation for common deletions in this study

**Additional file 5: Figure S4.** Long range effects of 7q11.1 inversion on chromatin conformation. A correlation heatmap shows chromatin interactions that are gained (red) and lost (blue) on the 7q11.1 inversion haplotype relative to the reference. The effect of the 7q11.1 inversion on chromatin conformation is similar to the effects of the 8p23.1 inversion, where the most dramatic effects involve contacts that span the inversion breakpoints. The inversion region is depicted by the black triangle

## Data Availability

Hi-C Contact Matrices by chromosome were deposited into NCBI’s Gene Expression Omnibus (accession GSE128678, https://bit.ly/2NbONMc), in conjunction with our companion study by Gorkin et al. [22]. Details and genotypes for common deletions are provided in the supplementary materials. The original structural variant calls can be downloaded directly at the following link: (ftp://ftp.1000genomes.ebi.ac.uk/vol1/ftp/phase3/integrated_sv_map/supporting/GRCh38_positions/) [10]. The eQTL calls can be downloaded from the supplementary material of Chiang et al. at the following link: (10.1038/ng.3834) [20]. The GWAS catalog can be downloaded directly from the web interface hosted at the NHGRI at the following link, which provides details about the file versions. This study used “All Associations v1.0.2” and the relevant study accession numbers are found within the file contents: (https://www.ebi.ac.uk/gwas/docs/file-downloads) [19].

## Abbreviations

AHUS: atypical Hemolytic Uremic Syndrome
DI: Directionality Index
EQTL: Expression Quantitative Trait Loci
LCLs: Lymphoblastoid Cell Lines
Non-TB: Not a TAD Boundary
OLSR: Ordinary Least Squares Regression
PCs: Principal Components
QQ: Quantile-Quantile
SDs: Segmental Duplications
SLE: Systemic Lupus Erythematosus
SV: Structural Variation
TAD: Topological Associating Domains
TB: TAD Boundary
λ: Genomic Inflation Factor

## References

[1] Lieberman-Aiden E, van Berkum NL, Williams L, Imakaev M, Ragoczy T, Telling A (2009). Comprehensive mapping of long-range interactions reveals folding principles of the human genome. Science.

[2] Dixon JR, Selvaraj S, Yue F, Kim A, Li Y, Shen Y (2012). Topological domains in mammalian genomes identified by analysis of chromatin interaction. Nature.

[3] McCord RP (2017). How to build a cohesive genome in 3d. Nature.

[4] Merkenschlager M, Nora EP (2016). Ctcf and cohesin in genome folding and transcriptional gene regulation. Annu Rev Genomics Hum Genet.

[5] Franke M, Ibrahim DM, Andrey G, Schwarzer W, Heinrich V, Schöpflin R (2016). Formation of new chromatin domains determines pathogenicity of genomic duplications. Nature.

[6] Goodman FR (2002). Limb malformations and the human hox genes. Am J Med Genet.

[7] Lupiáñez DG, Kraft K, Heinrich V, Krawitz P, Brancati F, Klopocki E (2015). Disruptions of topological chromatin domains cause pathogenic rewiring of gene-enhancer interactions. Cell.

[8] Sadowski M, Kraft A, Szalaj P, Wlasnowolski M, Tang Z, Ruan Y, Plewczynski D (2019). Spatial chromatin architecture alteration by structural variants in human genomes at the population scale. Genome Biol.

[9] Huynh L, Hormozdiari F (2019). TAD fusion score: discovery and ranking the contribution of deletions to genome structure. Genome Biol.

[10] Sudmant PH, Rausch T, Gardner EJ, Handsaker RE, Abyzov A, Huddleston J (2015). An integrated map of structural variation in 2,504 human genomes. Nature.

[11] Korbel JO, Urban AE, Affourtit JP, Godwin B, Grubert F, Simons JF (2007). Paired-end mapping reveals extensive structural variation in the human genome. Science.

[12] Cantsilieris S, Nelson BJ, Huddleston J, Baker C, Harshman L, Penewit K (2018). Recurrent structural variation, clustered sites of selection, and disease risk for the complement factor H (CFH) gene family. Proc Natl Acad Sci.

[13] Hughes AE, Orr N, Esfandiary H, Diaz-Torres M, Goodship T, Chakravarthy U (2006). A common CFH haplotype, with deletion of CFHR1 and CFHR3, is associated with lower risk of age-related macular degeneration. Nat Genet.

[14] Zhao J, Wu H, Khosravi M, Cui H, Qian X, Kelly JA (2011). Association of genetic variants in complement factor H and factor H-related genes with systemic lupus erythematosus susceptibility. PLoS Genet.

[15] Zipfel PF, Edey M, Heinen S, Józsi M, Richter H, Misselwitz J (2007). Deletion of complement factor H-related genes CFHR1 and CFHR3 is associated with atypical hemolytic uremic syndrome. PLoS Genet.

[16] Spielmann M, Lupiáñez DG, Mundlos S (2018). Structural variation in the 3D genome. Nat Rev Genet.

[17] Chaisson MJP, Sanders AD, Zhao X, Malhotra A, Porubsky D, Rausch T (2019). Multi-platform discovery of haplotype-resolved structural variation in human genomes. Nat Commun.

[18] Mohajeri K, Cantsilieris S, Huddleston J, Nelson BJ, Coe BP, Campbell CD (2016). Interchromosomal core duplicons drive both evolutionary instability and disease susceptibility of the chromosome 8p23.1 region. Genome Res.

[19] Welter D, MacArthur J, Morales J, Burdett T, Hall P, Junkins H (2013). The NHGRI GWAS catalog, a curated resource of SNP-trait associations. Nucleic Acids Res.

[20] Chiang C, Scott AJ, Davis JR, Tsang EK, Li X, Kim Y (2017). The impact of structural variation on human gene expression. Nat Genet.

[21] Lupiáñez DG, Spielmann M, Mundlos S (2016). Breaking TADs: how alterations of chromatin domains result in disease. Trends Genet.

[22] Gorkin DU, Qiu Y, Hu M, Fletez-Brant K, Liu T, Schmitt AD (2019). Common DNA sequence variation influences 3-dimensional conformation of the human genome. Genome Biol.

[23] Li H. Aligning sequence reads, clone sequences and assembly contigs with BWA-MEM. 2013;1303:3997.

[24] McVicker G, van de Geijn B, Degner JF, Cain CE, Banovich NE, Raj A (2013). Identification of genetic variants that affect histone modifications in human cells. Science.

[25] van de Geijn B, McVicker G, Gilad Y, Pritchard JK (2015). WASP: allele-specific software for robust molecular quantitative trait locus discovery. Nat Methods.

[26] Dixon JR, Jung I, Selvaraj S, Shen Y, Antosiewicz-Bourget JE, Lee AY (2015). Chromatin architecture reorganization during stem cell differentiation. Nature.

[27] Hu M, Deng K, Selvaraj S, Qin Z, Ren B, Liu JS (2012). HiCNorm: removing biases in hi-C data via Poisson regression. Bioinformatics.

[28] Purcell S, Neale B, Todd-Brown K, Thomas L, Ferreira MAR, Bender D (2007). PLINK: a tool set for whole-genome association and population-based linkage analyses. Am J Hum Genet.

[29] Peduzzi PN, Concato J, Kemper E, Holford TR, Feinstein AR (1996). A simulation study of the number of events per variable in logistic regression analysis. J Clin Epidemiol.

