# Supplementary figures and images for: The effects of common structural variants on 3D chromatin structure
Source: BMC Genomics. 2020 Jan 30;21:95. doi: 10.1186/s12864-020-6516-1 (PMC6990566; doi:10.1186/s12864-020-6516-1)

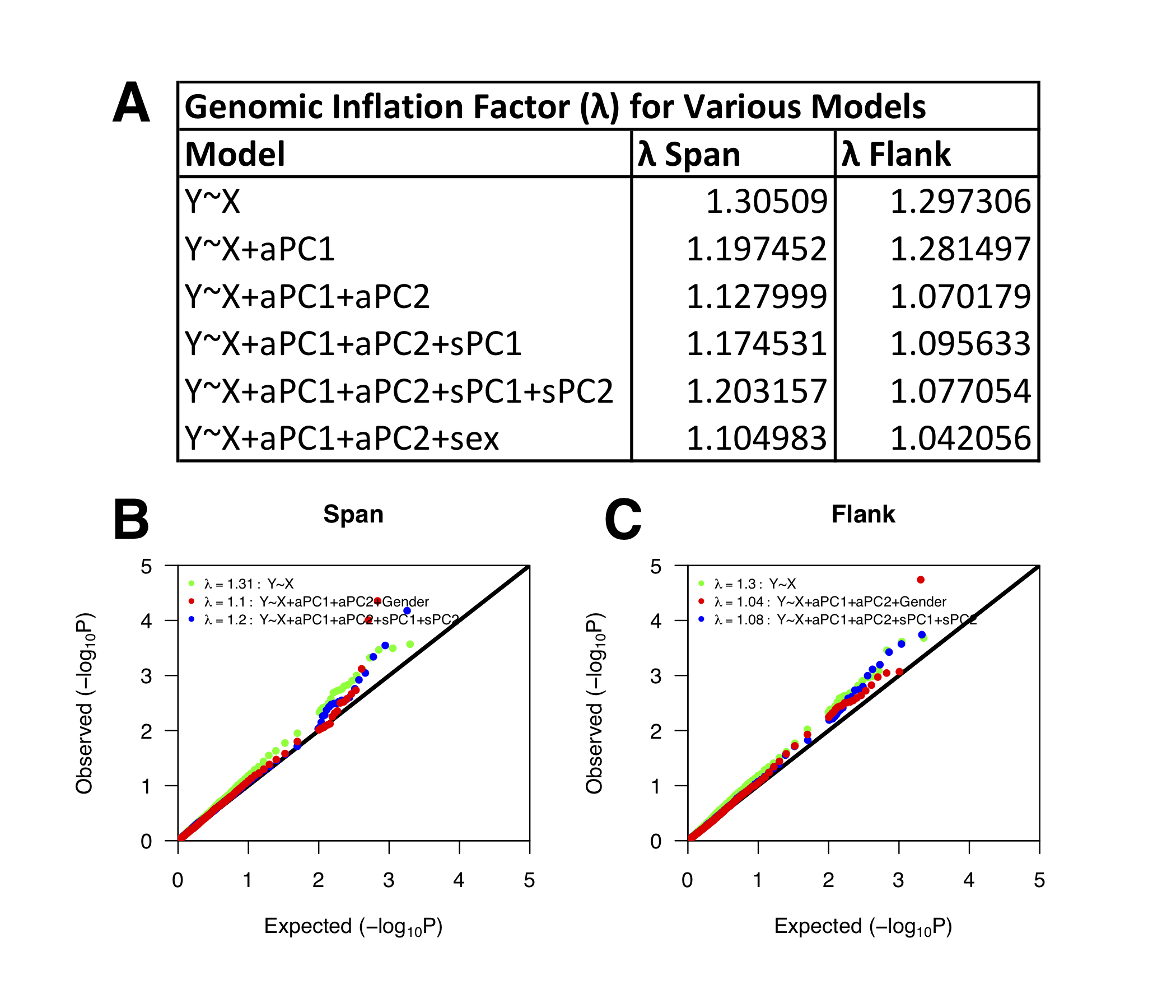

Supplement: Supplementary file 1 — Additional file 1: Figure S1. Ancestry principal components and sex need to be used as covariates in linear regression. To determine which covariates reduce the bias in the linear regression model, the effect of common deletions on chromatin conformation was tested for 6 different models, with each model adding an extra covariate term (Panel A). The genomic inflation factor was the metric used to determine the model with the least bias for possible confounding variables: ancestry principal components (PCs), sex, and surrogate variable PCs. The model that used ancestry PCs and sex as covariates had the least bias (λ = 1.10,1.04) and was chosen as the optimal model. P-values of the regression for each deletion in the span (Panel B) and flank (Panel C) region display how the chosen model still has inflation despite the low genomic inflation factor that can be attributed to real effects [file 12864_2020_6516_MOESM1_ESM.png]

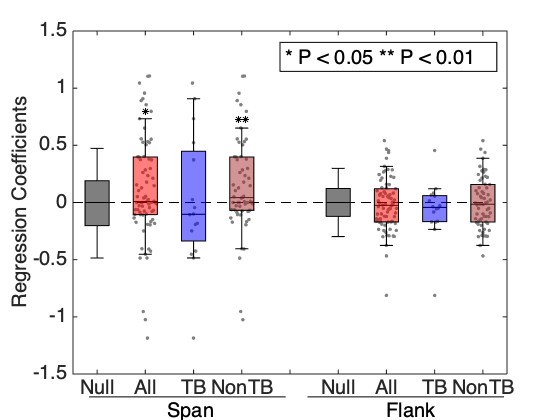

Supplement: Supplementary file 3 — Additional file 3: Figure S2. Masking segmental duplications does not change the effects of deletions on chromatin conformation. To determine if the effects on chromatin conformation are driven by segmental duplications (SD), a separate analysis was conducted for all large common deletions after masking every SD found within the deletion or in the flank regions. Deletions were stratified into groups of those that overlap with TAD boundaries (TB) and those that do not overlap with TAD boundaries (NonTB). A Wilcoxon rank-sum test was performed for each group against a null distribution and the results are consistent with the analysis that did not involve SD masking, showing that the effects of deletions on chromatin contacts are not driven by segmental duplications [file 12864_2020_6516_MOESM3_ESM.png]

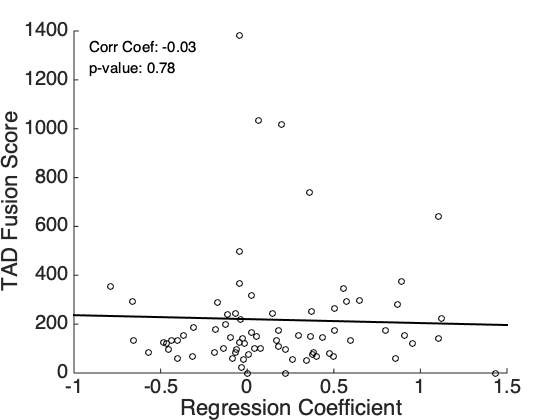

Supplement: Supplementary file 4 — Additional file 4: Figure S3. Linear regression coefficients in the span region do not correlate with TAD fusion score. We generated the TAD fusion score for our 80 large common deletions and compared the result with the linear regression coefficients in the span region. There was no significant correlation between the two different methods; suggesting that the fusion score is not predictive of patterns of chromatin conformation for common deletions in this study [file 12864_2020_6516_MOESM4_ESM.png]

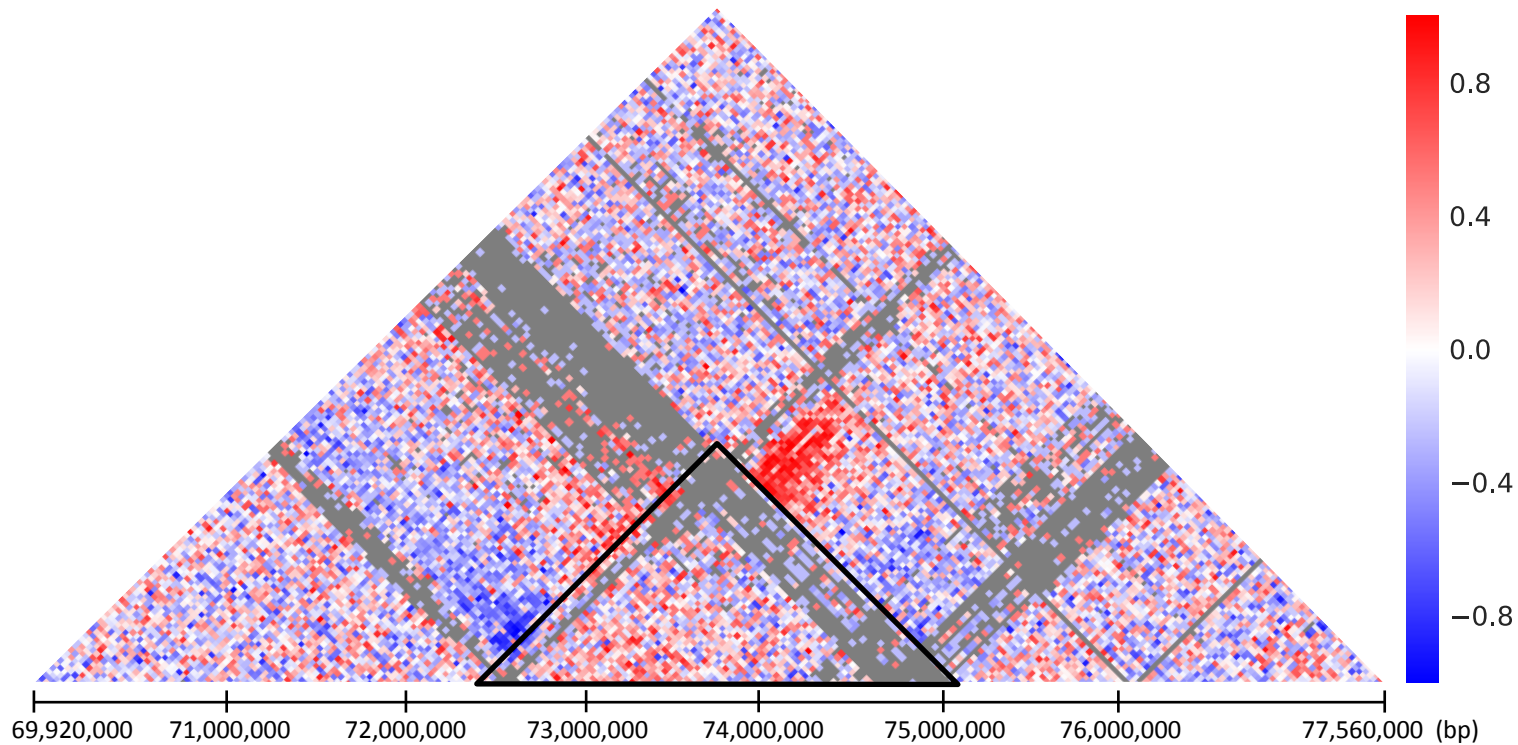

Supplement: Supplementary file 5 — Additional file 5: Figure S4. Long range effects of 7q11.1 inversion on chromatin conformation. A correlation heatmap shows chromatin interactions that are gained (red) and lost (blue) on the 7q11.1 inversion haplotype relative to the reference. The effect of the 7q11.1 inversion on chromatin conformation is similar to the effects of the 8p23.1 inversion, where the most dramatic effects involve contacts that span the inversion breakpoints. The inversion region is depicted by the black triangle [file 12864_2020_6516_MOESM5_ESM.pdf]
